# Digital gratification: short video consumption and mental health in rural China

**DOI:** 10.3389/fpubh.2025.1536191

**Published:** 2025-04-22

**Authors:** Chen Zhang, Bochen Zhu

**Affiliations:** Economics and Management School, Wuhan University, Wuhan, China

**Keywords:** short video, mental health, rural China, difference-in-differences, CFPS

## Abstract

**Background:**

In recent years, short videos have become increasingly popular in rural China, yet their impact on mental health remains underexplored. While prior studies have debated the psychological effects of social media, little is known about how short-form video consumption affects rural populations.

**Objective:**

This study investigates the causal relationship between short video consumption and mental health among rural residents in China.

**Methods:**

We use longitudinal data from the China Family Panel Studies and apply a Difference-in-Differences strategy to estimate the impact of frequent short video usage on mental health. To address self-selection and staggered treatment timing, we employ Propensity Score Matching and heterogeneity-robust difference-in-differences estimators. Robustness checks include placebo tests and an event study analysis.

**Results:**

We find that short video consumption appears to improve mental health among rural residents. The effect is immediate and significant only in the first year of exposure, but fades in subsequent periods. Mechanism analysis suggests that the improvements are driven by enhanced entertainment and information access rather than increased social interaction. The effects are more pronounced in economically underdeveloped and less pandemic-affected regions, but not evident among urban residents.

**Conclusion:**

Short videos provide short-term mental health benefits for rural Chinese residents by enriching their leisure and information access, especially in less developed areas. However, their positive effect is transient and cannot offset pandemic-related stress. Policy efforts should aim to balance the benefits of digital entertainment with potential risks such as addiction and information overload.

## 1 Introduction

For a long time, subjective wellbeing or happiness and its determinants in rural China have attracted great attention from economics, psychology, and other related disciplines. A number of studies suggest that there is an urban-rural disparity regarding subjective wellbeing due to socioeconomic inequality (1, 2). The existing scholarship has examined a range of factors that may affect rural dwellers' levels of life satisfaction, such as relative income, social capital, societal evaluations, health, and neighborhood infrastructure (3–6). Apart from these well-discussed elements, the dynamics of Chinese rural society may generate new drivers of the subjective wellbeing among rural residents. In recent years, a major attention-grabbing phenomenon in rural areas is the rapid popularity of short video applications like TikTok (or Douyin) and Kuaishou. Statistics show that as of June 2023, China has more than 1 billion short video users, 300 million of whom live in villages.^1^ Put differently, the penetration rate of short videos in rural areas is close to 60%. Meanwhile, given the huge market potential, many short video apps have further taken the countryside as the main direction for business expansion.^2^

Compared to their urban counterparts, rural dwellers in China lack enough leisure participation and ways to entertain themselves (7), which is detrimental to their happiness. Short videos can alleviate the disadvantages of rural people regarding daily entertainment choices. As an emerging media form, short video platforms have multiple functions, among which entertainment is particularly prominent (8). For example, approximately one-third of the top 30 most-viewed Chinese short videos from 2017 to 2018 were on the theme of pure entertainment.^3^ People in rural areas can receive, forward, comment, and even produce their own light-hearted and funny short video clips of various types on Kuaishou, Douyin, and other platforms. According to a survey conducted by the Chinese Academy of Social Sciences, more than 50% of the rural older adults take short videos as a major way of entertainment in their daily lives.^4^ On the contrary, for urban dwellers who have richer entertainment choices, short video usage may not significantly boost their happiness or mental health.

What impact, if any, does exposure to short videos have on the subjective wellbeing of rural residents? This paper addresses this question using panel data from the China Family Panel Studies (CFPS), a nationally representative survey, to examine whether frequent short video use improves mental health, as measured by the Center for Epidemiologic Studies Depression Scale (CES-D), where higher scores indicate poorer mental health. Since access to short video platforms is an endogenous choice, we employ a Difference-in-Differences (DID) approach to obtain credible estimates. The DID method exploits the panel structure by comparing individuals over time (within-group variation), while the validity of between-group comparisons relies on the parallel pre-treatment trends between treatment and control groups.

Short video consumption is a self-selected behavior shaped by factors such as education and socioeconomic status, making non-users an inappropriate counterfactual group. To address this, we employ Propensity Score Matching (PSM) (9) to pre-process the data before applying a Difference-in-Differences (DID) strategy. While our baseline analysis uses the traditional Two-Way Fixed Effects (TWFE) model, its validity may be compromised under staggered treatment timing. Given that individuals may both start and stop using short videos over time, we further adopt the heterogeneity-robust estimators developed by De Chaisemartin and d'Haultfoeuille (10) to ensure more reliable inference.

Baseline regressions show that frequent short video use significantly reduces CES-D scores among rural residents, indicating improved mental health, whereas no such effect is found for urban populations. These results are robust to the use of PSM-DID and heterogeneity-robust estimators. Event study analyses confirm parallel pre-trends and suggest that the mental health benefit is immediate but fades over time with continued use. Additional robustness checks, including placebo tests and controls for other online activities, further validate the findings.

To explore potential mechanisms, we find that high-frequency short video users increasingly value the internet for entertainment and information access, while no changes are observed for work or socializing, pointing to leisure and information as key pathways. Moreover, the positive effect is stronger in less developed regions and areas less severely affected by the pandemic, suggesting that short videos help alleviate everyday stress but are less effective under acute psychological pressure.

This study speaks to literature exploring the relationship between internet use and mental health. Previous studies have highlighted the complex, multi-layered impact of internet use on mental health. For instance, while internet use among students can provide social support, it can also lead to increased anxiety and stress due to information overload (11). Similarly, older adults benefit from social activities online that alleviate depression and improve wellbeing, but excessive information can negatively affect satisfaction (12). Digital finance development enhances connections with friends and relatives, reducing depression (13). Additionally, internet addiction in high-stress groups, such as medical residents, often exacerbates anxiety and lowers self-esteem (14). Building on this foundation, our research focuses on short videos as a newer internet phenomenon that has rapidly gained global influence, spurring widespread interest and even geopolitical controversy, including U.S. discussions of regulatory bans (15), highlighting its complex, multi-dimensional cultural and political impacts (16).

Our paper also speaks to research on the social impacts of short videos, including effects on culture, consumer behavior, and mental health. Existing literature suggests that short videos play a crucial role in cultural transmission and value shaping, especially among young people, by conveying positive social values but also, in some cases, fostering an entertainment-first orientation that may be misleading (17). Additionally, short videos show significant effects in advertising and consumer behavior, stimulating purchasing desire through social attributes (18). The frequent use of short videos also introduces risks of attention fragmentation and addiction, with addicted users showing notable declines in cognitive function and focus (19). Different from prior research, our study centers on short video use among rural residents in China. Short video platforms in China are often regarded as having effectively captured users in the “sinking market”, which refers to areas such as towns and rural regions (20, 21). However, the psychological effects on these groups, who are central to the platform's success in China, remain underexplored (22).

The remainder of this paper is organized as follows. Section 2 develops the theoretical hypotheses. Section 3 introduces the data and identification strategies. Section 4 presents the main regression results along with a series of robustness checks. Section 5 discusses the findings in relation to previous literature. Section 6 concludes the paper with policy implications, limitations, and suggestions for future research.

## 2 Theoretical hypotheses

Little research has investigated the causal relationship between short video consumption and people's happiness, but the relevant influence of the internet, particularly social media, has been widely discussed over the past two decades (23, 24). However, the findings drawn by prior studies are somewhat contradicted. Prior literature indicates that social media plays a dual role, potentially inducing stress while simultaneously providing coping mechanisms and supportive resources (25). Some studies based on data in China and overseas reveal that frequent use of social media is positively associated with affective polarization, depression, and other symptoms of poor mental health [e.g., (26–30)]. On the contrary, many other studies find that access to the internet and social media contributes to people's mental health by expanding and maintaining social connections and efficiently consuming online entertainment content [e.g., (31, 32)]. Under this circumstance, the impact of short videos, as a new type of internet media, on individuals' mental health and happiness may be positive or negative. Therefore, this study hypothesizes:

**H 1a**. *Frequent short video consumption has a positive impact on Chinese rural residents' mental health*.

**H 1b**. *Frequent short video consumption has a negative impact on Chinese rural residents' mental health*.

Short videos typically feature easily digestible content, including humorous skits, daily life vlogs, music, and dance, which cater to a wide range of preferences and literacy levels, and serve multiple functions such as entertainment, information access, social interaction, and even income generation (33–35). These functions suggest that short videos may influence mental health through multiple channels. Previous studies have shown that entertainment and information access positively impact stress reduction and anxiety alleviation (36, 37). The impact of social interactions on mental health is ambivalent. On the one hand, they can enhance psychological wellbeing by offering social support (38, 39); on the other hand, they may provoke negative emotions through social comparison, thereby undermining mental health (26). In addition, income generation contributes positively to mental health (40). On this basis, the following hypothesis is formulated:

**H 2**. *Short videos affect rural residents' mental health via their functions of entertainment, information access, social interaction, and income generation*.

The emergence of short video consumption coincided with the COVID-19 pandemic in China, leading to the likelihood that short videos may have played a crucial role during this challenging time. Short videos might have improved mental health especially for those living in regions severely affected by the pandemic. This hypothesis arises from the unique nature of the pandemic, which brought about widespread lockdowns, restricted mobility, and increased anxiety, forcing individuals to find new means of coping with stress and staying connected with others (41). Specifically, short videos might provide convenient entertainment that helped alleviate stress during the pandemic, particularly in rural areas where entertainment options were limited (42, 43). Additionally, they facilitated virtual social connections, reducing loneliness, and served as a source of practical information, such as pandemic updates and health advice, which helped manage uncertainty and anxiety (44, 45). However, another possibility is that the observed positive effects of short video consumption on mental health are independent of the pandemic context. Short video platforms inherently provide opportunities for entertainment and information, which can be beneficial for mental health even outside of a crisis. Therefore, it is important to disentangle whether the positive effects are unique to the context of the pandemic or if they are due to the inherent attributes of short-form videos. In other words, we aim to examine whether the pandemic serves as a prerequisite for the effectiveness of short video consumption. Thus, we hypothesize that:

**H 3a**. *Short videos have a positive impact on mental health in regions severely affected by the pandemic*.

**H 3b**. *Short videos have a positive impact on mental health in regions less affected by the pandemic*.

## 3 Methods

### 3.1 Data

To examine the causal relationship between short video consumption and subjective wellbeing, we use data from the China Family Panel Studies (CFPS), a nationally representative biennial survey initiated in 2010 that covers a wide range of personal health topics and is widely used in health economics research (46, 47). All family members identified at baseline are permanently tracked. The CFPS includes questions based on the widely adopted self-reported CES-D scale, which measures depressive symptoms in the general population. Given that short videos are a relatively recent phenomenon, CFPS only began collecting data on short video usage in 2020 and 2022, whereas CES-D data are available for 2012, 2016, 2018, 2020, and 2022. Due to limited rural internet infrastructure before 2016, short video applications were not widely accessible. Although platforms began targeting the “sinking market” (i.e., rural regions) in 2018, the 2018 CFPS did not include short video usage data. We therefore exclude the 2018 wave and focus on the 2012, 2016, 2020, and 2022 waves. The sample is further restricted to individuals who consistently resided in rural areas, resulting in a final sample of 5,053 respondents after data cleaning.

### 3.2 Variable definition and description

The CES-D (Center for Epidemiologic Studies Depression Scale) was developed by the National Institute of Mental Health in 1977 to assess the frequency and severity of depressive symptoms in the general population. The original 20-item version (CES-D20) covers key dimensions of depression, including depressed mood, guilt, worthlessness, helplessness, psychomotor retardation, appetite loss, and sleep disturbance. Respondents rate symptom frequency over the past week on a 1-to-4 scale, yielding a total score ranging from 8 to 80, where higher scores indicate greater depression severity. CFPS adopted the CES-D20 in 2012 and, for improved survey efficiency, switched to a shortened 8-item version (CES-D8) in 2016, which retains core components of the original scale. To ensure score comparability across waves, CFPS applied the equipercentile equating method to harmonize CES-D8 and CES-D20, generating a unified score labeled CESD20sc that maintains the original CES-D20 range. Following CFPS guidelines, we use the CESD20 score as the primary outcome variable to ensure cross-wave comparability. In regression analyses, we normalize this score within each survey wave using the z-score.

The key independent variable is respondents' short video consumption, measured as a binary indicator equal to 1 if a respondent reported watching short videos almost every day, and 0 otherwise. We exploit the limited 4G network access in rural China prior to the launch of the China Universal Telecom Service Program in 2016 (48), which provides a quasi-natural experiment setting for our difference-in-differences (DID) design. Survey waves from 2016 and earlier serve as the pre-treatment period, while respondents reporting daily short video use in or after 2020 constitute the treatment group. In our sample, 1,032 individuals began using short videos in 2020, 720 in 2022, and 347 started in 2020 but later exited. This variation in treatment timing creates a staggered adoption structure with both entry and exit, for which we apply a heterogeneity-robust DID estimator to obtain more reliable estimates.

Demographic controls include gender, age, Han ethnicity, years of schooling, CCP membership, family size, household income per capita, marital status, and job type, following (49). The regression analysis incorporates province-year fixed effects to account for regional and temporal variations, and for robustness, we add provincial-level covariates such as GDP per capita, rural population share, rural per capita disposable income, and rural broadband penetration. Individual-level variables are drawn from CFPS, while provincial-level data come from the China County Statistical Yearbook. Table 1 reports summary statistics by short video usage status, showing that frequent users tend to have lower CES-D20 scores, are younger, and have more years of education than non-users.

**Table 1:** Summary statistics.

	**Non-frequent users**		**Frequent users**	
**Variables**	**Mean**	**SD**	**Mean**	**SD**
	**(1)**	**(2)**	**(3)**	**(4)**
CES-D20 score	33.397	8.354	32.637	7.537
Male	0.588	0.492	0.546	0.498
Age	52.619	14.110	40.581	13.342
Han	0.911	0.285	0.916	0.277
**Marriage**				
Unmarried	0.059	0.236	0.151	0.358
Married or living together	0.884	0.320	0.816	0.388
Divorced or widowed	0.057	0.232	0.033	0.179
Years of schooling	7.964	2.724	9.219	2.759
CCP member	0.102	0.303	0.090	0.286
Family size	4.354	2.024	4.552	1.872
Per capita househould income	8.956	1.173	9.257	1.115
**Employment**				
Unemployed	0.215	0.411	0.143	0.350
Employed	0.759	0.428	0.785	0.411
Student	0.026	0.158	0.071	0.257

### 3.3 Empirical strategy

In this study, we employ a Difference-in-Differences (DID) approach with a standard two-way fixed effects model to evaluate the impact of frequent short video consumption on individuals' mental health. The empirical model is represented by the following equation:


(1) $$\begin{array}{c} Y_{i,t,p}=\alpha_{0}+\alpha_{1}Short\;video\;consumption_{i,t}+\alpha_{2}{\text{X}}_{i,t}+\lambda_{i}\\ +\mu_{t}+\nu_{p,t}+\varepsilon_{i,t,p} \end{array}$$

where *Y*_*i, t, p*_ represents the normalized CES-D score for individual *i* surveyed in province *p* during year *t* of the CFPS. The variable *Short video consumption*_*i, t*_ takes the value of 1 if individual *i* watched short videos daily. The coefficient of interest, α_1_, measures the effect of daily short video consumption on the outcome *Y*_*i, t, p*_ during the treatment periods. The vector **X**_*i, t*_ includes demographic variables listed in Table 1. We control for individual fixed effects, λ_*i*_, to ensure comparisons are within individuals, capturing the difference before and after adopting short videos in daily life. We also include year-specific fixed effects, μ_*t*_, and province-year fixed effects, ν_*p, t*_, to account for systematic differences shared by individuals in the same regions and surveyed in the same year. These controls help tease out the impact of macro-level socio-economic variations associated with provinces and survey years. Additionally, we replace the province-year fixed effects with time-varying provincial characteristics to test the robustness of our findings. The error term is represented by ε_*i, t, p*_, and standard errors are clustered at the individual level for robust inference.

## 4 Results

Table 2 presents the baseline regression results. We incrementally add covariates across columns. Column (1) controls only for the basic two-way fixed effects, Column (2) adds demographic controls, Column (3) further incorporates provincial-level covariates, and Column (4) replaces these provincial-level covariates with province-year fixed effects as specified in Equation 1. For time-invariant variables, such as gender and ethnicity, we interact these variables with year dummies to control for differential time trends across groups defined by these characteristics. The results indicate that frequent short video consumption significantly reduces CES-D scores among rural residents by approximately 0.05 standard deviations, supporting Hypothesis 1a over Hypothesis 1b. With the exception of Column (1), all results are statistically significant at the 5% level, suggesting that short videos can alleviate depressive symptoms to some extent for rural residents. In contrast, Table 3 replicates the regression analysis from Table 2, with the only difference being the exclusive use of urban residents in the sample. We find that short video consumption has no significant impact on the mental health of urban residents, as the regression coefficients across all columns are insignificant and close to zero.

**Table 2:** Baseline estimates of short video consumption's impact on mental health.

	**(1)**	**(2)**	**(3)**	**(4)**
Short video consumption	-0.037^*^	-0.059^***^	-0.055^**^	-0.053^**^
	(0.027)	(0.030)	(0.030)	(0.030)
Demographics	No	Yes	Yes	Yes
Provincial controls	No	No	Yes	No
Individual FE	Yes	Yes	Yes	Yes
Year FE	Yes	Yes	Yes	Yes
Province-year FE	No	No	No	Yes
#Obs	25,058	16,872	16,782	16,870
#Clusters	7,601	5,053	5,037	5,053
Adjusted R-squared	0.418	0.430	0.430	0.439

OLS estimators are reported in this table. Standard errors clustered at the individual level are reported in parentheses. ^*^, ^**^, and ^***^ represent significance at 10, 5, and 1% levels, respectively.

**Table 3:** Comparison analysis using urban sample.

	**(1)**	**(2)**	**(3)**	**(4)**
Short video consumption	0.013	0.009	0.004	0.010
	(0.027)	(0.030)	(0.030)	(0.030)
Demographics	No	Yes	Yes	Yes
Provincial controls	No	No	Yes	No
Individual FE	Yes	Yes	Yes	Yes
Year FE	Yes	Yes	Yes	Yes
Province-year FE	No	No	No	Yes
#Obs	20,549	17,066	15,919	17,063
#Clusters	6,000	4,943	4,738	4,943
Adjusted R-squared	0.436	0.444	0.444	0.449

OLS estimators are reported in this table. Standard errors clustered at the individual level are reported in parentheses. ^*^, ^**^, and ^***^ represent significance at 10, 5, and 1% levels, respectively.

According to data from the China Time Use Survey (CTUS), in 2017, prior to the advent of the short video era, urban residents in China spent an average of 4.71 h per day on leisure activities, compared to 4.52 h for rural residents. The difference in total leisure time between urban and rural residents was not significant; however, their allocation of leisure time differed noticeably. For instance, urban residents spent an average of 0.69 hours on physical exercise, compared to 0.39 hours for rural residents. Similarly, urban residents spent 0.34 h on reading, while rural residents spent only 0.17 h. Urban residents generally devoted twice as much time to more proactive leisure activities as their rural counterparts, suggesting that they had more leisure options.

Given that short videos are designed for vertical screens on mobile devices, we compared mobile internet usage between urban and rural residents. According to CFPS data used in this study, in 2016, 51% of urban residents accessed the internet via mobile devices, compared to just 32% of rural residents. By 2020, the proportion of urban residents using mobile internet had risen to 72%, with an average daily usage of 3.18 hours, while the proportion in rural areas exceeded 50%, reaching 56%, with an average daily usage of 2.58 hours. Regarding short video adoption, by 2020, 84% of rural mobile internet users had engaged with short videos, higher than the 79% in urban areas. Additionally, the proportion of daily short video app users was 57% in rural areas, significantly higher than 53% in urban areas. This suggests that although urban residents use mobile devices for internet access more frequently, they engage less with short video apps compared to rural residents, indicating that urban residents likely allocate more time to other online activities.

These findings provide indirect evidence for the urban-rural differences in short video usage observed in the baseline results. Despite urban residents having more entertainment options and better internet coverage, rural residents utilized short videos more frequently after their emergence. This suggests that short videos occupy a significant niche in the leisure activities of rural residents, which partly explains why short videos significantly improve the mental health of rural residents but have a relatively weaker impact on urban residents.

However, as shown in Table 1, there are systematic differences between frequent short video users and non-users in rural areas. To make the control and treatment groups more comparable, we use Propensity Score Matching to identify suitable matches for the treated individuals before re-conducting the DID analysis.

### 4.1 PSM-DID

In the following matching process, we use 1:1 nearest neighbor matching to identify the most similar counterpart for each treated individual. Specifically, to avoid common PSM errors, such as matching individuals from different years or matching different time points for the same individual, we utilize cross-sectional data at the individual level, treating different years of the same variable in the panel dataset as distinct variables. Only pre-treatment variables are used in the matching process.

Due to the entry and exit dynamics within the treatment group, we categorize treated individuals into three subgroups: those who used short videos in both 2020 and 2022, those who used them only in 2020, and those who used them only in 2022. We separately identify the best matching counterparts for each of these three subgroups from the control group, and aggregate the matching weights accordingly. For individuals who used short videos in both 2020 and 2022, as well as those who used them only in 2020, we use their demographic information from 2016 or earlier for matching. For individuals who began using short videos only in 2022, demographic information from 2020 is also included in the matching process.

**Figure 1:**
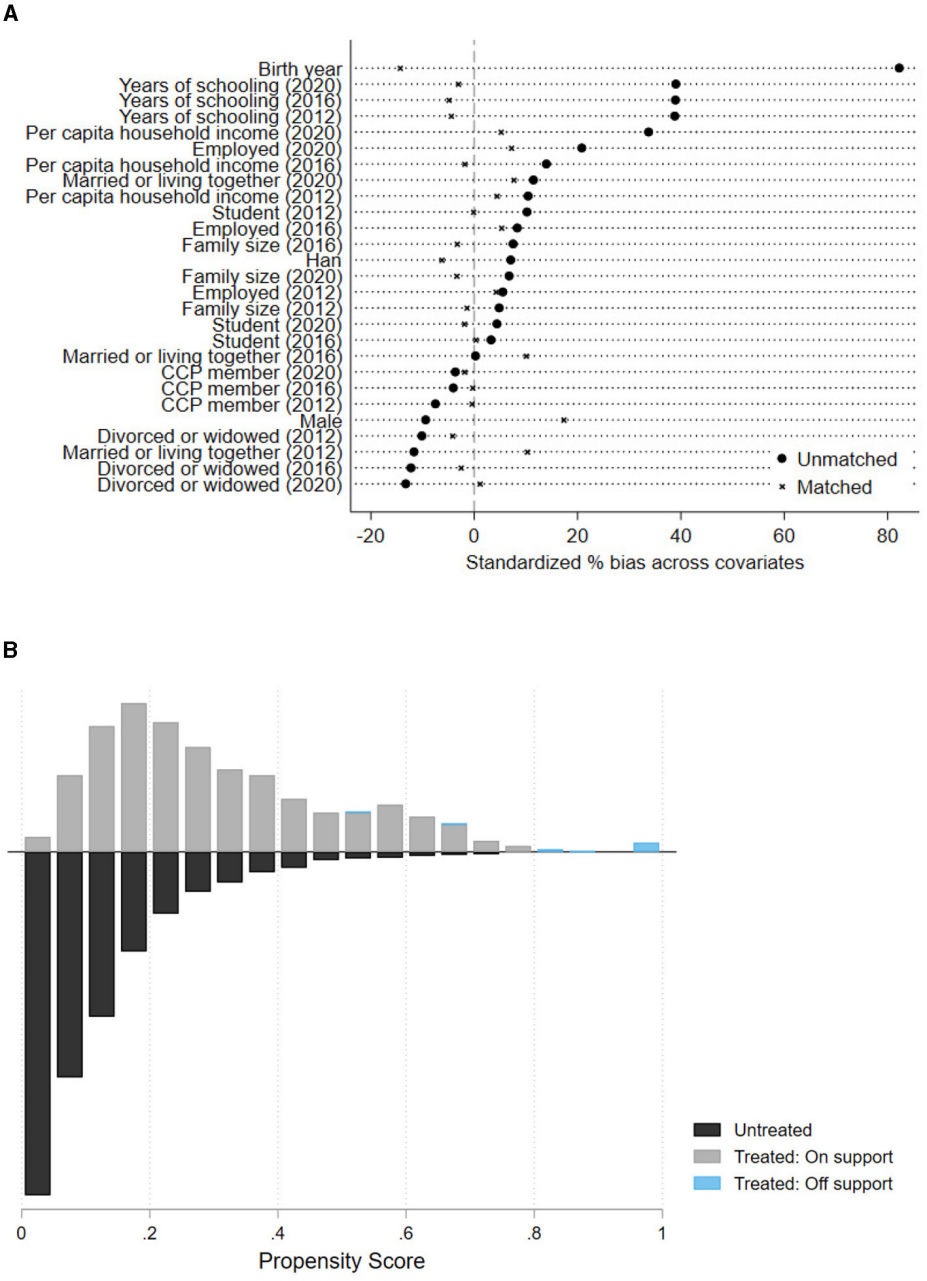
Balance and overlap diagnostics for PSM. **(A)** Covariate standardization bias test. **(B)** Common support area.

Table 4 presents the results of the covariate balance tests. These balance tests allow us to assess the extent to which differences in covariates between the treatment and control groups have been reduced, helping us determine whether the matched sample approximates random selection. The column labeled “% bias” in the table shows the bias scores for covariates before and after matching. Overall, all variables have bias scores below the 20% threshold. Additionally, the last two columns display the t-test results for differences between the treatment and control groups before and after matching. Except for gender and birth year, all covariates exhibit no significant differences after matching. Figure 1A visually depicts the balance of covariates before and after matching, with most variables clustering around zero. Therefore, after matching, our treatment and control groups are generally well-balanced, approximating random grouping.

**Table 4:** Balancing of covariates.

	**Unmatched**	**Mean**			**%reduct**	***t*** **-test**	
**Variable**	**Matched**	**Treated**	**Control**	**%bias**	**Bias**	**t**	***p*** **> t**
Male	U	0.544	0.590	-9.4		-2.94	0.003
	M	0.606	0.520	17.3	-84.1	2.58	0.01
Birth year	U	1974.3	1964.1	82.2		25.23	0
	M	1972.5	1974.3	-14.3	82.6	-2.27	0.023
Han	U	0.927	0.908	7.1		2.18	0.029
	M	0.913	0.930	-6.3	11.2	-1	0.316
**Marriage (2012)**							
Married or living together	U	0.874	0.910	-11.7		-3.71	0
	M	0.886	0.855	10.3	12	1.37	0.17
Divorced or widowed	U	0.019	0.036	-10.1		-3.06	0.002
	M	0.015	0.022	-4.2	58.6	-0.71	0.475
**Marriage (2016)**			
Married or living together	U	0.909	0.908	0.2		0.08	0.939
	M	0.924	0.895	10	-3984.6	1.45	0.148
Divorced or widowed	U	0.029	0.053	-12.2		-3.7	0
	M	0.023	0.028	-2.6	79	-0.47	0.639
**Marriage (2020)**			
Married or living together	U	0.917	0.892	8.3		2.55	0.011
	M	0.924	0.909	5.3	36.9	0.81	0.416
Divorced or widowed	U	0.044	0.075	-13.2		-3.99	0
	M	0.042	0.039	1.1	91.7	0.2	0.843
Years of schooling (2012)	U	8.781	7.812	38.8		12.03	0
	M	8.708	8.820	-4.5	88.4	-0.69	0.49
Years of schooling (2016)	U	8.926	7.876	38.9		12.13	0
	M	8.849	8.981	-4.9	87.4	-0.73	0.464
Years of schooling (2020)	U	9.097	8.066	39		12.12	0
	M	9.057	9.139	-3.1	92.1	-0.46	0.646
CCP member (2012)	U	0.070	0.090	-7.5		-2.31	0.021
	M	0.080	0.081	-0.5	93.8	-0.07	0.945
CCP member (2016)	U	0.089	0.101	-4		-1.25	0.21
	M	0.095	0.096	-0.3	91.8	-0.05	0.96
CCP member (2020)	U	0.100	0.111	-3.6		-1.13	0.257
	M	0.106	0.112	-1.9	48.4	-0.28	0.783
Family size (2012)	U	4.632	4.546	4.8		1.48	0.138
	M	4.561	4.586	-1.4	70.3	-0.23	0.82
Family size (2016)	U	4.583	4.435	7.5		2.33	0.02
	M	4.527	4.592	-3.3	55.9	-0.5	0.619
Family size (2020)	U	4.461	4.323	6.8		2.07	0.038
	M	4.464	4.533	-3.4	49.5	-0.5	0.619
Per capita household income (2012)	U	8.669	8.550	10.4		3.25	0.001
	M	8.757	8.707	4.4	58	0.7	0.481
Per capita household income (2016)	U	8.842	8.678	14		4.37	0
	M	8.843	8.865	-1.9	86.7	-0.29	0.768
Per capita household income (2020)	U	9.664	9.368	33.7		10.14	0
	M	9.619	9.574	5.2	84.7	0.82	0.411
**Employment (2012)**							
Employed	U	0.664	0.638	5.5		1.73	0.084
	M	0.678	0.658	4.2	24.4	0.63	0.526
Student	U	0.030	0.015	10.2		3.32	0.001
	M	0.038	0.038	-0.2	98.3	-0.02	0.984
**Employment (2016)**							
Employed	U	0.888	0.849	11.5		3.53	0
	M	0.920	0.895	7.6	33.3	1.28	0.2
Student	U	0.011	0.008	3.3		1.04	0.297
	M	0.015	0.015	0.3	91.2	0.04	0.972
**Employment (2020)**							
Employed	U	0.905	0.835	20.8		6.29	0
	M	0.920	0.896	7.2	65.5	1.2	0.229
Student	U	0.002	0.000	4.4		1.47	0.141
	M	0.000	0.001	-1.9	56.5	-0.41	0.678

**Figure 2:**
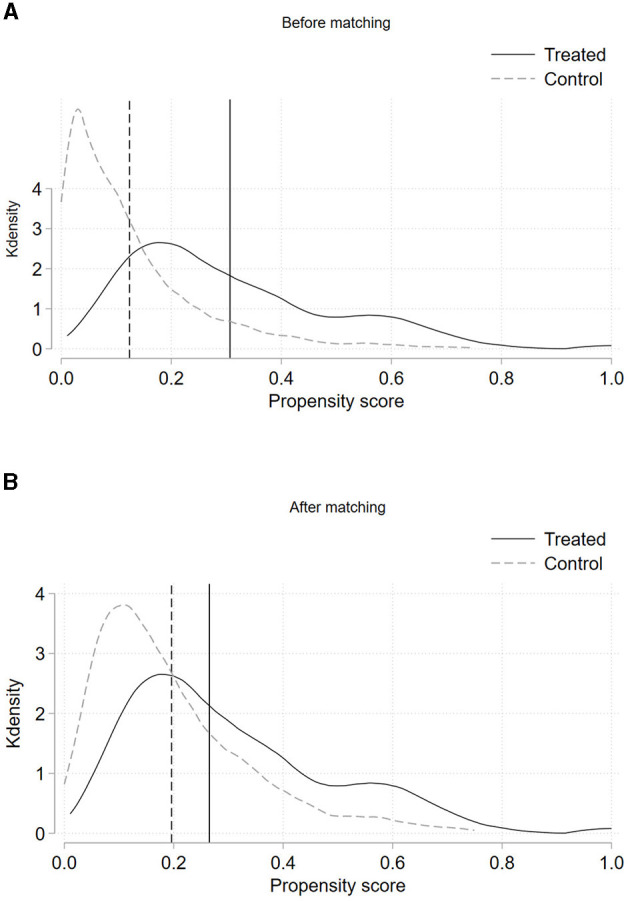
Density comparisons before and after matching. **(A)** Density map of pre-treatment group and control group. **(B)** Matched density map of post-treatment group and control group.

Figure 1B illustrates the support for the common support assumption in the matching results. The majority of treated group samples were successfully matched, with only 21 samples being off support. Figure 2 displays the density curves for the treatment and control groups before and after matching, respectively. We observe that the kernel density curves after matching are closer and smoother compared to those before matching. These tests indicate that the matching is successful, providing a solid foundation for re-estimating the treatment effect using the DID analysis.

Next, we re-estimate the results obtained in Table 2 using the matched sample and weights. The specifications of each regression in Table 5 are identical to those in Table 2. We find that after applying PSM, the significance of the key independent variable, short video consumption, improves. Except for the coefficients in Column (3), which are significant at the 5% level, all other coefficients are significant at the 1% level. Additionally, the magnitude of the estimated coefficients increases to approximately 0.085. This suggests that the baseline estimates were likely underestimated due to differences between the treatment and control groups.

**Table 5:** PSM-DID estimation.

	**(1)**	**(2)**	**(3)**	**(4)**
Short video consumption	-0.084^***^	-0.086^***^	-0.083^**^	-0.086^***^
	(0.041)	(0.041)	(0.041)	(0.041)
Demographics	No	Yes	Yes	Yes
Provincial controls	No	No	Yes	No
Individual FE	Yes	Yes	Yes	Yes
Year FE	Yes	Yes	Yes	Yes
Province-year FE	No	No	No	Yes
#Obs	9,225	9,111	9,086	9,110
#Clusters	2,464	2,460	2,459	2,460
Adjusted R-squared	0.391	0.393	0.393	0.408

OLS estimators are reported in this table. Standard errors clustered at the individual level are reported in parentheses. ^*^, ^**^, and ^***^ represent significance at 10, 5, and 1% levels, respectively.

However, PSM resulted in a loss of about half of the sample, which is a limitation of PSM–potentially significant sample attrition. Therefore, we consider the PSM-DID results as supplementary and supportive evidence for the robustness of our baseline findings. More importantly, the results of the parallel trends test play a crucial role. If the pre-treatment trends between the treatment and control groups are parallel, it indicates that even if there are systematic differences between the two groups, the post-treatment effect can still be attributed to changes brought about by short video consumption. The specific results are discussed in Section 4.3.

### 4.2 Heterogeneity-robust DID estimator

Another challenge in identifying the impact of short video consumption is the instability of user behavior, given that short videos are a relatively new phenomenon. Individuals start using short videos at different times, and some users may even discontinue usage later. Usually, DID approaches treat treatment status as static, assuming stable distinctions between treatment and control groups. However, directly using high-frequency short video consumption as the DID variable while employing a standard two-way fixed effects (TWFE) model can introduce several issues in this context.

When treatment timing is staggered, using the standard TWFE model can lead to biased estimates due to its inability to account for treatment heterogeneity over time. Specifically, TWFE assumes that treatment effects are homogeneous across units and time, which does not hold in situations where individuals begin and end treatment at different times. This limitation can result in negative weighting issues and unreliable effect estimates, especially if the treatment status varies dynamically. To address these issues, we employ the heterogeneity-robust estimator proposed by De Chaisemartin and d'Haultfoeuille (10). Their estimator is well-suited for staggered treatment structures and can accommodate cases where individuals enter or exit the treatment group.

The estimation results are presented in Table 6. All coefficients are significant and slightly higher than the baseline estimates, indicating a reduction in CES-D scores by 0.054–0.068 standard deviations. The difference between the heterogeneity-robust estimator and the TWFE estimates is minimal, likely because negative weighting was not a major issue in our TWFE model. An analysis of the weights in Column (4) of Table 2 reveals that 475 out of 3,103 weights are negative, accounting for 15.3% of the total, but only 3.95% when considering the magnitude of the weights. This suggests that while there may be some bias in the baseline results, it is not severe.

**Table 6:** Heterogeneity-robust estimation.

	**(1)**	**(2)**	**(3)**	**(4)**
ATT	-0.046^*^	-0.068^**^	-0.063^**^	-0.054^*^
	(0.026)	(0.030)	(0.030)	(0.031)
Demographics	No	Yes	Yes	Yes
Provincial controls	No	No	Yes	No
Individual FE	Yes	Yes	Yes	Yes
Year FE	Yes	Yes	Yes	Yes
Province-year FE	No	No	No	Yes

OLS estimators are reported in this table. Standard errors clustered at the individual level are reported in parentheses. ^*^, ^**^, and ^***^ represent significance at 10, 5, and 1% levels, respectively.

### 4.3 Event study

The core assumption of the DID approach is the parallel trends assumption. This assumption is crucial for ensuring the validity of the DID model, as it implies that in the absence of treatment, the treatment and control groups would have followed the same trend over time. Satisfying the parallel trends assumption suggests that any differences observed between the treatment and control groups after treatment can be attributed to the treatment itself, rather than to pre-existing differences or divergent trends.

We use an event study approach to dynamically illustrate the effects before and after treatment across each period. Before 2016, there were almost no short video users in rural areas, allowing us to compare differences between individuals who would eventually use short videos and those who did not, prior to 2016. Specifically, we adopt the specification of Equation 1, but replace the DID variable with a series of interaction terms between time indicators relative to treatment timing and short video consumption. The interaction terms do not include the period just before individuals began using short videos, and this period works as the benchmark period.

**Figure 3:**
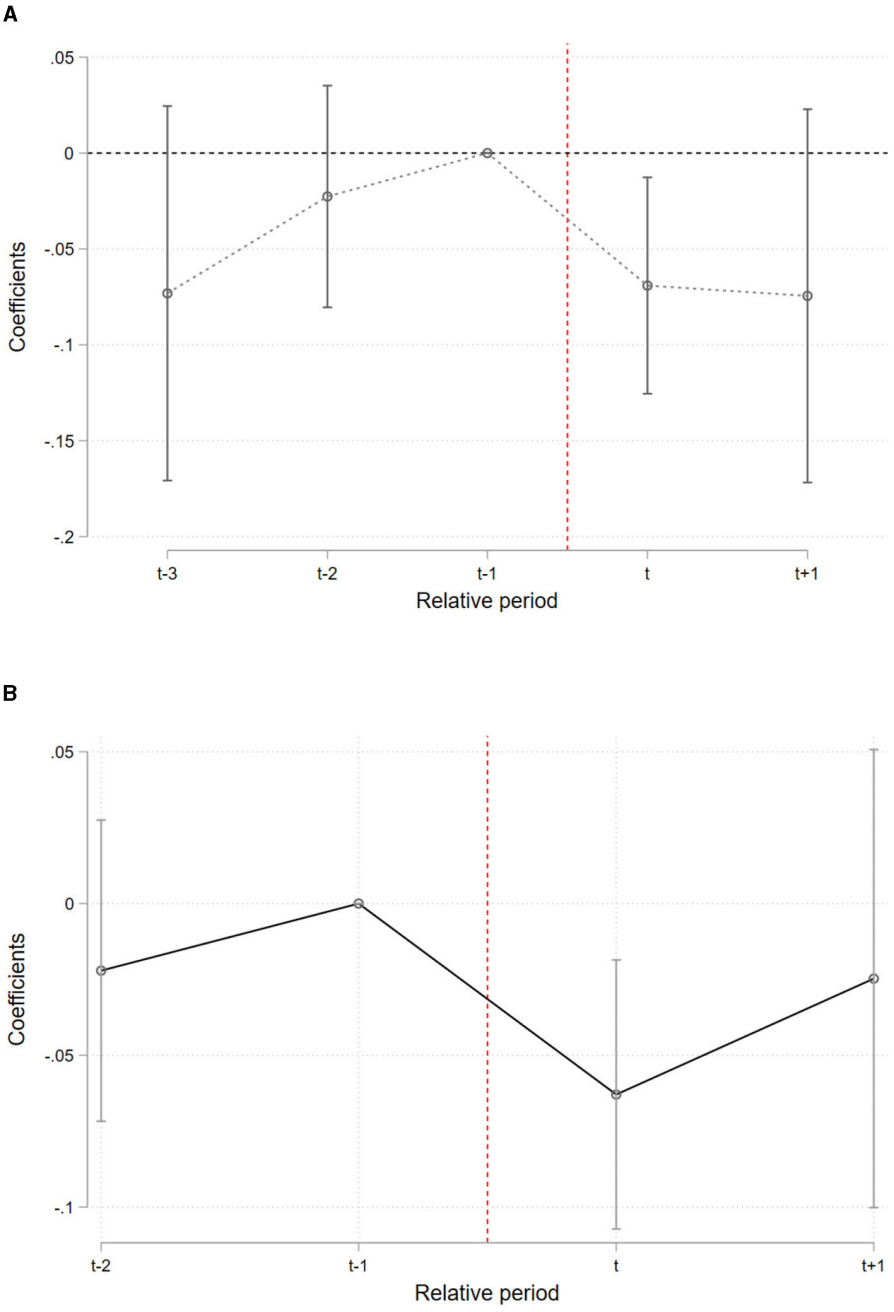
Dynamic effects of treatment: event study results. **(A)** TWFE. **(B)** Heterogeneity-robust estimators.

As shown in Figure 3A, taking individuals who began watching short videos only in 2022 as an example, the year 2012 represents the *t*−3 period for them. The results in De Chaisemartin and d'Haultfoeuille indicate that, prior to short video consumption, there were no significant differences between the treatment and control groups. However, in the year of first exposure to short videos, the CES-D score for the treatment group significantly decreased by 0.07 standard deviations, which is greater than the average post-treatment effect estimated in the baseline analysis. Nevertheless, the effect of short videos appears to be temporary, as it becomes insignificant in the subsequent period.

Additionally, Figure 3B shows the dynamic effects estimated using the heterogeneity-robust estimator proposed by De Chaisemartin and d'Haultfoeuille (10). A notable aspect of their estimator is that, when estimating dynamic effects, it limits the number of pre-treatment periods to not exceed the number of post-treatment periods. Taking those watching short videos only in 2022 as an example, for them, 2020 is the first pre-treatment period. Thus, for this group, the pre-treatment placebo effects can only be estimated up to 2016, which is why Figure 3B has one fewer pre-treatment period compared to Figure 3A. The results in Figure 3B are consistent with those in Figure 3A, with significant effects observed only in the initial period. Compared to the baseline results, the event study approach provides a more detailed view of the short video effects over time, demonstrating that the impact of short videos is immediate but not persistent.

### 4.4 Placebo tests

**Figure 4:**
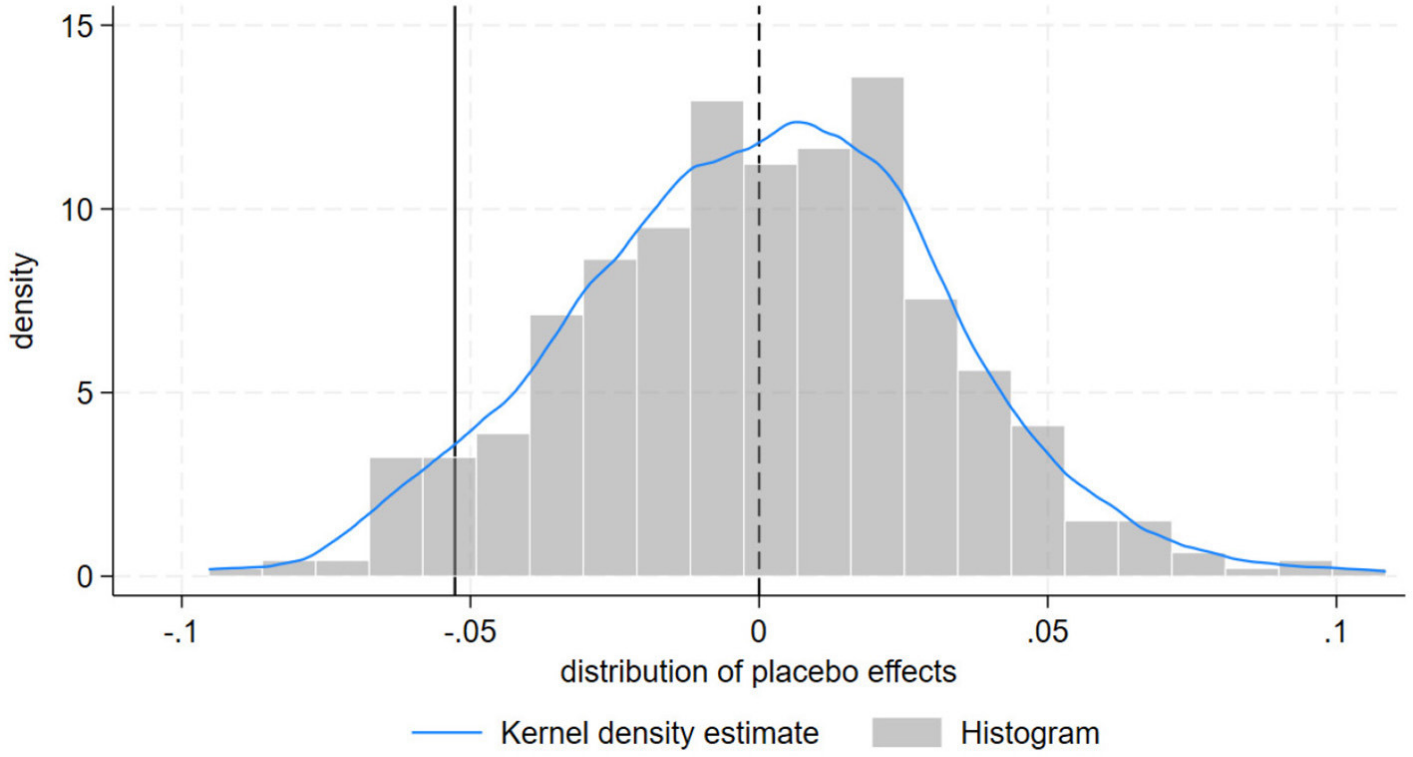
Mixed placebo test.

We conduct two types of placebo tests. First, a permutation test is to assess the robustness of our findings. By randomly assigning treatment times or treatment units, we can evaluate whether the observed treatment effects are due to chance rather than the actual intervention. This type of test strengthens the credibility of our results by ensuring that the effects are not merely artifacts of the data or the modeling approach. Specifically, we conduct mixed placebo tests by randomly assigning placebo treatment timings and placebo treatment units, repeating the baseline regression 500 times. The distribution of estimated placebo coefficients of DID item are presented in Figure 4. We observe that the actual estimated value falls at the 5.6% percentile, which is below 10%, indicating that our baseline results pass the placebo test. This demonstrates that our results are not due to chance.

The second placebo test we conduct is related to other online activities. Some may wonder whether the positive emotional effects of short video consumption are attributable to other forms of internet use. This is a reasonable concern, as those who adopt new technologies like short videos may also be heavy internet users, engaging in online shopping, social networking, and other forms of entertainment simultaneously.

To address this concern, we adopt a method commonly known as “horse racing,” where other online activities are included in the regression model to see if they account for the observed effects. Specifically, CFPS contains information on online gaming, online shopping, and online learning. In Table 7, we sequentially add these variables into the regression in Columns (1) through (3), and in Column (4), we include all other online activities simultaneously. The other specifications are consistent with Equation 1.

**Table 7:** Horse racing comparison by controlling for other online activities.

	**(1)**	**(2)**	**(3)**	**(4)**
Short video consumption	-0.054^**^	-0.057^**^	-0.046^**^	-0.051^**^
	(0.022)	(0.022)	(0.022)	(0.022)
Online games	0.029			0.030
	(0.052)			(0.052)
Online shopping		0.156^*^		0.165^**^
		(0.084)		(0.084)
Online learning			-0.131^***^	-0.137^***^
			(0.045)	(0.045)
Demographics	Yes	Yes	Yes	Yes
Individual FE	Yes	Yes	Yes	Yes
Year FE	Yes	Yes	Yes	Yes
Province-year FE	Yes	Yes	Yes	Yes
#Obs	16,870	16,870	16,870	16,870
#Clusters	5,053	5,053	5,053	5,053
Adjusted R-squared	0.439	0.439	0.439	0.439

OLS estimators are reported in this table. Standard errors clustered at the province-schooling cohort level are reported in parentheses. ^*^, ^**^, and ^***^ represent significance at 10, 5, and 1% levels, respectively.

We find that even after including these other activities, the coefficient for short video consumption remains significant, with magnitudes similar to the baseline results. This suggests that the impact of short video consumption on mental health is not confounded by whether individuals are also engaged in other online activities. The horse racing method increases the credibility of our regression results by directly testing competing explanations for the observed effects. If the improved mental health were driven by overall internet usage, we would expect the inclusion of these variables to diminish the significance of short video consumption. The fact that the effect remains robust indicates that it is indeed short video consumption, rather than other online activities, that is driving the improvements in mental health.

### 4.5 Potential mechanisms

The CFPS is the only large-scale survey in China that includes questions on the frequency of short video app usage. However, the survey's exploration of short videos is limited to this aspect and does not inquire about the types of videos watched by respondents. As a result, we cannot determine which specific type of short videos improves the mental health of rural residents. What is known is that most content offered by short video platforms is entertainment-oriented. Fortunately, the CFPS provides respondents' evaluations of the importance of the internet for various purposes, including work, socializing, entertainment, and information access. Specifically, respondents rated the importance of these functions using a 5-point Likert scale. If a respondent values a specific function more after using short videos compared to others, it can be inferred that short videos contribute to that function. Based on this, we explore the relationship between such functions and mental health as discussed in the literature.

We conducted regression analysis using the baseline specification of Equation 1, with the only difference being that the dependent variables were replaced by respondents' ratings of the importance of the internet for work, socializing, entertainment, and information access (scored on a scale from 1 to 5). The results are presented in Table 8. Compared to the trend observed in the control group, high-frequency consumption of short videos significantly increased respondents' ratings of the importance of the internet for entertainment and information access. Surprisingly, despite being a form of social media, short videos did not enhance respondents' evaluations of the importance of internet-based socializing. This may be due to the weak emphasis on familiar social connections in short video content. Literature also suggests that social media can lead to social comparison, particularly among acquaintances, which may negatively affect mental health (27, 50). Therefore, we conclude that the provision of entertainment content and the expansion of information access channels are potential mechanisms through which short videos alleviate negative emotions, while socializing and work may not play a significant role. These findings partially lend support to Hypothesis 2.

**Table 8:** Potential mechanisms.

**Dep. Var**.	**Importance of … when using the internet**			
	**Working**	**Socializing**	**Entertainment**	**Information access**
	**(1)**	**(2)**	**(3)**	**(4)**
Short video consumption	0.052	0.024	0.137^***^	0.376^***^
	(0.056)	(0.039)	(0.047)	(0.050)
Demographics	Yes	Yes	Yes	Yes
Individual FE	Yes	Yes	Yes	Yes
Year FE	Yes	Yes	Yes	Yes
Province-year FE	Yes	Yes	Yes	Yes
#Obs	4,905	6,472	6,396	12,693
#Clusters	2,204	2,486	2,473	4,557
Adjusted R-squared	0.305	0.336	0.272	0.505

OLS estimators are reported in this table. Standard errors clustered at the individual level are reported in parentheses. ^*^, ^**^, and ^***^ represent significance at 10, 5, and 1% levels, respectively.

### 4.6 Heterogeneity analysis

To verify which of these hypotheses holds, we conducted a heterogeneity analysis to explore the differential effects of short video consumption based on the severity of the pandemic in different regions. Based on the severity of the pandemic, we classified provinces with a median or higher number of cases per 100,000 people during the 2020-2022 period as high-severity regions and those below the median as low-severity regions. We then re-estimated the effect of short video consumption on mental health separately for these two categories of regions. This approach allows us to determine whether the observed effects varied depending on the pandemic's impact on the respective regions, thereby providing insights into the contextual dependence of short video effects.

**Figure 5:**
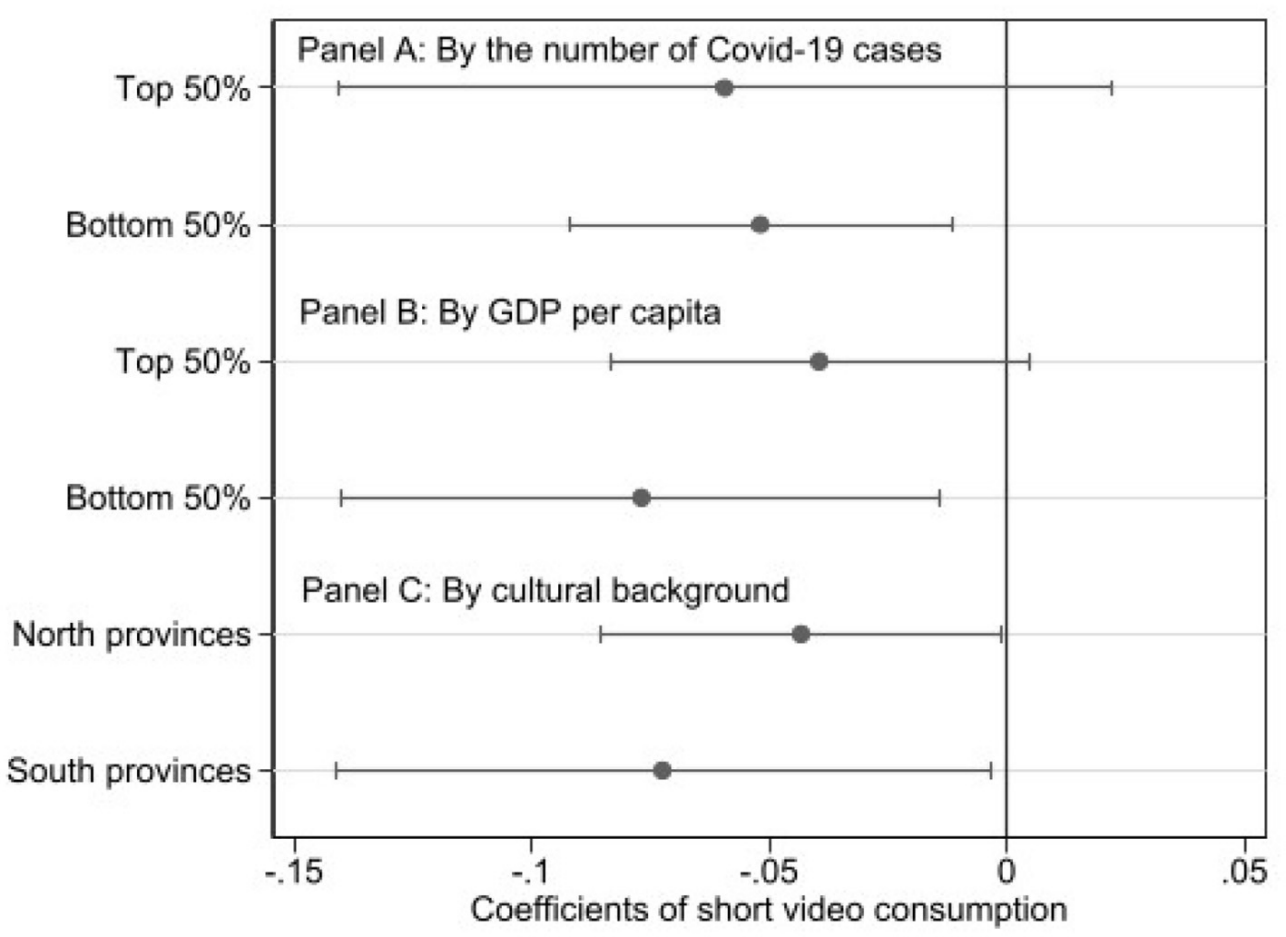
Heterogeneous effect.

The results, presented in Panel A of Figure 5, indicate that in less severely affected regions, short video consumption significantly alleviated negative emotions, with significance levels and coefficient magnitudes comparable to the baseline results. In contrast, no significant causal effect was found in regions more severely affected by the pandemic. These findings provide support for Hypothesis 3b rather than Hypothesis 3a. In regions where the pandemic did not create significant additional stressors, the role of short videos in promoting mental health is likely tied to their inherent attributes–providing joy and a means of information access. In contrast, in high-severity regions, where individuals were likely dealing with higher levels of stress, anxiety, and possibly even grief, the impact of short videos was not significant. This suggests that while short videos can contribute to wellbeing in normal circumstances, their capacity to offset severe stress and anxiety, such as that brought about by the pandemic, is limited.

Additionally, we examined the heterogeneity of short video effects based on economic development and cultural differences. In Panel B of Figure 5, provinces were divided into two groups based on the median per capita GDP, and the effects of short video consumption were re-estimated within each group. The results indicate that short videos have a more pronounced positive impact on mental health in economically underdeveloped rural areas, echoing our previous findings. Baseline results show that short videos significantly influence rural samples but have no significant effect in urban samples. Furthermore, provinces with relatively mild pandemic impacts are often characterized by lower population mobility and weaker economic development, suggesting that less diversified entertainment markets, or “sinking market,” are where short videos exert their positive effects. Notably, this consumer group has been largely overlooked in previous research. Finally, we find no significant differences in the effects of short videos between northern and southern provinces in Panel C of Figure 5.

## 5 Discussion

In this study, we address the question of whether short video consumption impacts mental health among rural residents in China. Using a DID approach, we aimed to identify a causal relationship between frequent short video usage and mental health improvements. The analysis used longitudinal data from the CFPS, focusing on the CES-D depression scale as a measure of mental wellbeing. Our findings indicate that short video consumption is associated with immediate mental health benefits, particularly effective in the first period of usage but showing diminishing effects thereafter. Additionally, these mental health improvements are more significant in regions with lower pandemic severity and weaker economic development, suggesting that short videos provide meaningful leisure but cannot fully mitigate pandemic-related stress.

Our findings contribute to the growing literature on the relationship between short video use and mental health, while extending it in important ways. Existing studies have primarily focused on adolescents and the older adults. For example, a study based on interviews and grounded theory found that college students in Henan Province with psychological disorders and insufficient social support tend to use short videos to satisfy unmet social needs (51). Another study utilizing the same CFPS dataset as ours focused on older adults and showed that short video use improves their mental health by enhancing intergenerational relationships and leisure consumption (52). However, other research has highlighted potential risks. A cross-lagged panel network analysis of a youth sample in China revealed a significant association between short video addiction and depressive symptoms (53). Similarly, survey-based research on college students in the Arabian Gulf region found a negative correlation between short video addiction and mental health, particularly in the context of platforms like Instagram Reels and YouTube Shorts (54). Compared to these studies, which emphasize either specific age groups or the addictive use of short videos, our study highlights the potential mental health benefits of short video consumption for rural residents–a relatively underexplored population. Additionally, recent work has shown that embedding mental health content into short videos can help reduce stigma, alleviate anxiety and depression, and offer psychological support during public health crises, especially among adolescents in China and Japan (34, 55–58).

For rural areas lacking in entertainment infrastructure, short videos can serve as a temporary means of alleviating negative emotions. However, the effects of short video consumption are transient. This may stem from their recommendation algorithms, and solutions to extend their positive impact should also be found at the algorithmic level. Personalized recommendation algorithms collect users' interests, preferences, and behavioral data (e.g., recommendation techniques based on previous content choices or social connections) (59). These algorithms free users from an overload of irrelevant information, transforming the process from “people search for information” to “information finds people.” However, the greedy recommendation features of such algorithms can lead to information narrowing, redundancy, and overload, which in turn may cause user fatigue and psychological resistance (60). In our data, nearly 10% of users reduced or stopped using short videos after 2020. This could explain why the positive impact of short videos on mental health is not sustained.

Through the analysis of potential mechanisms, we found that frequent short video viewing enhances individuals' recognition of the importance of entertainment and information acquisition in online activities. Existing literature also highlights the positive effects of entertainment and information acquisition on mental health. However, the ways short video platforms are used vary greatly. From the perspective of user behavior, usage can be categorized into actively uploading videos and passively watching them. In terms of video formats, it can be divided into regular short videos and more interactive live-streaming videos. Regarding content, besides the dominant entertainment-oriented videos, there are also educational videos and lifestyle videos with social attributes. Thus, the mechanism analysis in this study is exploratory, and specific content and formats of short videos may have heterogeneous effects on mental health. Some cutting-edge studies have made relevant attempts. For instance, experiments delivering motivational and comedic content to college students have been shown to improve their mental health (61). Similarly, recent surveys reveal that actively uploading videos enhances young users' subjective wellbeing more than passively watching, while viewing content focused on people and fashion, compared to entertainment content, significantly decreases subjective wellbeing (62).

## 6 Conclusion

The policy implications of this study should be interpreted with caution. The potential risk of addiction to short videos has been widely criticized. Studies have shown that the longer users engage with short video applications, the clearer their personal profiles become, with accumulated behavioral data further strengthening the personalized recommendation algorithms. This mechanism immerses users in a passive information-receiving state and subjects them to invisible control, ultimately leading to addiction to short videos (63). Moreover, research indicates that viewing personalized recommended short videos continually stimulates the brain's ventral tegmental area (VTA), which is responsible for pleasure and motivational reinforcement. Prolonged activation of this area leads to cravings and eventual addiction (64). The literature also suggests that addiction to short videos can be observed in both younger and older populations (65, 66). Addictive behaviors reduce social willingness and increase feelings of loneliness, with loneliness and addiction reinforcing each other, thereby negatively impacting mental health (34, 65).

Therefore, we suggest a multi-level intervention strategy to mitigate the potential mental health risks associated with short video use. At the platform level, recommendation algorithms should be optimized to go beyond homogeneous entertainment content and incorporate more beneficial and positive material. For rural residents, in particular, platforms could provide targeted recommendations and popular science content that align with their cultural background and living environment, thereby improving mental health awareness. Furthermore, when users encounter content that may trigger negative emotions–such as material that encourages social comparison or exacerbates social tensions–the system could recommend counterbalancing emotional content to foster a healthier digital environment. Platforms may also adopt “nudge” techniques, such as setting viewing time reminders or encouraging breaks, to prevent excessive use. At the community level, local governments should actively promote digital literacy and mental health education among rural residents. In addition, investing in leisure infrastructure and organizing cultural and skill-building activities can help reduce overreliance on short videos by providing alternative sources of engagement and social interaction. Overall, this paper is not intended to promote short video platforms, but to underscore the importance of enriching the cultural and recreational lives of rural residents.

Despite the robustness of our empirical strategy and the consistency of results across various checks, this study has several limitations that should be acknowledged. First, the CFPS dataset only records the frequency of short video use but does not provide detailed information about the content, format, or engagement type (e.g., active creation vs. passive consumption), which constrains our ability to identify more nuanced mechanisms. Second, while the study accounts for individual fixed effects and applies matching techniques, there may still be unobserved time-varying factors influencing both short video usage and mental health, which cannot be fully ruled out. Third, the mental health effect identified in this study is short-term, and the long-term psychological consequences of habitual short video consumption remain unclear. Future research could benefit from combining survey data with digital trace data to better capture content characteristics and user behavior patterns. Additionally, experimental designs could help establish stronger causal inferences regarding specific types of video content or recommendation algorithms. Investigating the interplay between digital literacy, media trust, and mental health outcomes across different demographic groups also presents a promising avenue for further study.

## Data Availability

Publicly available datasets were analyzed in this study. This data can be found here: the China Family Panel Studies (CFPS) database, https://www.isss.pku.edu.cn/cfps/en/.

## Footnotes

^1^Data source: https://www.cnnic.net.cn/NMediaFile/2023/0807/MAIN169137187130308PEDV637M.pdf.

^2^See, for example, *36Kr*. 13 January 2022. “The End of Short Videos Leads to the ‘Rural' Landscape.” https://36kr.com/p/1554841233772417.

^3^Data source: http://paper.people.com.cn/xwzx/html/2018-09/01/content_1908380.htm.

^4^Data source: https://www.sohu.com/a/641941430_433965.

## References

[1] Asadullah MN Xiao S Yeoh E. Subjective well-being in China, 2005-2010: the role of relative income, gender, and location. China Econ Rev. (2018) 48:83–101. 10.1016/j.chieco.2015.12.010

[2] Liang Y Niu X Lu P. The aging population in China: subjective well-being of empty nesters in rural eastern China. J Health Psychol. (2020) 25:361–72. 10.1177/1359105317717599 28810487

[3] Cai S Wang J. Less advantaged, more optimistic? Subjective well-being among rural, migrant and urban populations in contemporary. China China Econ Rev. (2018) 52:95–110. 10.1016/j.chieco.2018.06.005

[4] Clark WA Yi D Huang Y. Subjective well-being in China's changing society. Proc Nat Acad Sci. (2019) 116:16799–804. 10.1073/pnas.1902926116 31371499 PMC6708306

[5] Han C. Explaining the subjective well-being of urban and rural Chinese: income, personal concerns, and societal evaluations. Soc Sci Res. (2015) 49:179–90. 10.1016/j.ssresearch.2014.08.006 25432612

[6] Yip W Subramanian SV Mitchell AD Lee DT Wang J Kawachi I. Does social capital enhance health and well-being? Evidence from rural China. Soc Sci Med. (2007) 64:35–49. 10.1016/j.socscimed.2006.08.027 17029692

[7] Chen N Tsai CTL. Rural-urban divide and the social stratification in leisure participation in China: application of multiple hierarchy stratification perspective. Appl Res Qual Life. (2020) 15:1535–48. 10.1007/s11482-019-09750-z

[8] Zhang Z. Infrastructuralization of Tik Tok: Transformation, power relationships, and platformization of video entertainment in China. Media, Cult Soc. (2021) 43:219–36. 10.1177/0163443720939452

[9] Heckman JJ Ichimura H Todd PE. Matching as an econometric evaluation estimator: Evidence from evaluating a job training programme. Rev Econ Stud. (1997) 64:605–54. 10.2307/2971733

[10] De Chaisemartin C d'Haultfoeuille X. Difference-in-differences estimators of intertemporal treatment effects. In: Review of Economics and Statistics. (2024) p. 1–45. 10.1162/rest_a_01414

[11] Abellaneda JB Malbas MH Moneva J Navarroza MW. Internet use and mental health among students. J Stud Educ. (2022) 13:1–8. 10.5296/jse.v13i1.20518

[12] Lam SSM Jivraj S Scholes S. Exploring the relationship between internet use and mental health among older adults in england: longitudinal observational study. J Med Intern Res. (2019) 22:15683. 10.2196/preprints.15683 32718913 PMC7420689

[13] Liao L Du M. How digital finance shapes residents' health: evidence from China. China Econ Rev. (2024) 87:102246. 10.1016/j.chieco.2024.10224638145072

[14] Ueno T Ito K Murai T Fujiwara H. Mental health problems and their association with internet use in medical residents. Front Public Health. (2020) 8:587390. 10.3389/fpubh.2020.587390 33194994 PMC7641600

[15] Gray J. The geopolitics of ‘platforms': the TikTok challenge. Internet Policy Rev. (2021) 10:1557. 10.14763/2021.2.1557

[16] Miao W Huang D Huang Y. More than business: the de-politicisation and re-politicisation of TikTok in the media discourses of China, America and India (2017–2020). Media Int Austral. (2021) 186:97–114. 10.1177/1329878X211013919

[17] Guo Q. The influence of short video on college students' values from the perspective of new media. In: 2020 5th International Conference on Mechanical, Control and Computer Engineering (ICMCCE). (2020). p. 2414–2417. 10.1109/ICMCCE51767.2020.00520

[18] Ge J Sui Y Zhou X Li G. Effect of short video ads on sales through social media: the role of advertisement content generators. Int J Advert. (2021) 40:870–96. 10.1080/02650487.2020.1848986

[19] Chen Y Li M Guo F Wang X. The effect of short-form video addiction on users' attention. Behav Inform Technol. (2022) 42:2893–910. 10.1080/0144929X.2022.2151512

[20] Li M Tan CK Yang Y. Shehui Ren: cultural production and rural youths' use of the Kuaishou video-sharing app in Eastern China. Inform Commun Soc. (2020) 23:1499–514. 10.1080/1369118X.2019.1585469

[21] Wang W Wu J. Research perspectives on TikTok & its legacy apps| short video platforms and local community building in China. Int J Commun. (2021) 15:23.

[22] Zhang C Zheng H Wang Q. Driving factors and moderating effects behind citizen engagement with mobile short-form videos. IEEE Access. (2022) 10:40999–1009. 10.1109/ACCESS.2022.3167687

[23] Bell V. Online information, extreme communities and internet therapy: Is the internet good for our mental health? J Mental Health. (2007) 16:445–57. 10.1080/09638230701482378

[24] O'Keeffe GS Clarke-Pearson K. The impact of social media on children, adolescents, and families. Pediatrics. (2011) 127:800–4. 10.1542/peds.2011-0054 21444588

[25] Wolfers LN Utz S. Social media use, stress, and coping. Curr Opini Psychol. (2022) 45:101305. 10.1016/j.copsyc.2022.101305 35184027

[26] Allcott H Braghieri L Eichmeyer S Gentzkow M. The welfare effects of social media. Am Econ Rev. (2020) 110:629–76. 10.1257/aer.20190658

[27] Braghieri L Levy R Makarin A. Social media and mental health. Am Econ Rev. (2022) 112:3660–93. 10.1257/aer.20211218

[28] Golin M. The effect of broadband Internet on the gender gap in mental health: evidence from Germany. Health Econ. (2022) 31:6–21. 10.1002/hec.4570 35833231

[29] McDool E Powell P Roberts J Taylor K. The internet and children's psychological wellbeing. J Health Econ. (2020) 69:102274. 10.1016/j.jhealeco.2019.102274 31887480

[30] Yue Z Zhang R Xiao J. Passive social media use and psychological well-being during the COVID-19 pandemic: the role of social comparison and emotion regulation. Comput Human Behav. (2022) 127:107050. 10.1016/j.chb.2021.107050 34646057 PMC8499034

[31] Lu H Kandilov I. Does mobile internet use affect the subjective well-being of older Chinese adults? An instrumental variable quantile analysis. J Happin Stud. (2021) 22:3137–56. 10.1007/s10902-021-00365-635564970

[32] Pénard T Poussing N Suire R. Does the Internet make people happier? J Socio-Econ. (2013) 46:105–16. 10.1016/j.socec.2013.08.004

[33] Alvarenga P. Cerezo MÁ, Wiese E, Piccinini CA. Effects of a short video feedback intervention on enhancing maternal sensitivity and infant development in low-income families. Attachm Human Dev. (2020) 22:534–54. 10.1080/14616734.2019.1602660 30961424

[34] Yao Y She K Wang Y. Deconstructing social contact: short video-mediated internet addiction in the post-COVID-19 era (a research survey based on university students). Curr Psychol. (2025) 2025:1–17. 10.1007/s12144-025-07293-1

[35] Zhang X Wu Y Liu S. Exploring short-form video application addiction: socio-technical and attachment perspectives. Telematics Inform. (2019) 42:101243. 10.1016/j.tele.2019.101243

[36] Powell J Clarke A. Internet information-seeking in mental health: population survey. Br J Psychiat. (2006) 189:273–7. 10.1192/bjp.bp.105.017319 16946364

[37] Oliver MB Raney AA. Entertainment as pleasurable and meaningful: Identifying hedonic and eudaimonic motivations for entertainment consumption. J Commun. (2011) 61:984–1004. 10.1111/j.1460-2466.2011.01585.x

[38] Harandi TF Taghinasab MM Nayeri TD. The correlation of social support with mental health: a meta-analysis. Elect Physi. (2017) 9:5212. 10.19082/5212 29038699 PMC5633215

[39] Turner RJ Brown RL. Social support and mental health. In: A Handbook for the Study of Mental Health: Social Contexts, Theories, and Systems. Cambridge: Cambridge University Press (2010). p. 200–12.

[40] Patel V. Mental health in low-and middle-income countries. Br Med Bull. (2007) 81:81–96. 10.1093/bmb/ldm010 17470476

[41] Nimrod G. Changes in internet use when coping with stress: older adults during the COVID-19 pandemic. Am J Geriat Psychiat. (2020) 28:1020–4. 10.1016/j.jagp.2020.07.010 32771312 PMC7372257

[42] Ganter SA. Young adults' perceptions of entertainment consumption in their everyday lives during the COVID-19 pandemic: negotiating versatility, emotions, and agency in times of limited choice. Media, Cult Soc. (2024) 2024:01634437241291467. 10.1177/01634437241291467

[43] Xu Y Wang J Ma M. Adapting to lockdown: exploring stress coping strategies on short video social media during the COVID-19 pandemic. Psychol Res Behav Managem. (2023) 16:5273–87. 10.2147/PRBM.S441744 38170068 PMC10759421

[44] Chen Q Min C Zhang W Ma X Evans R. Factors driving citizen engagement with government TikTok accounts during the COVID-19 pandemic: model development and analysis. J Med Internet Res. (2021) 23:e21463. 10.2196/21463 33481756 PMC7864626

[45] Southwick L Guntuku SC Klinger EV Seltzer E McCalpin HJ Merchant RM. Characterizing COVID-19 content posted to TikTok: public sentiment and response during the first phase of the COVID-19 pandemic. J Adolesc Health. (2021) 69:234–41. 10.1016/j.jadohealth.2021.05.010 34167883 PMC8217440

[46] Liao L Du M Chen Z. Environmental pollution and socioeconomic health inequality: evidence from China. Sustain Cities Soc. (2023) 95:104579. 10.1016/j.scs.2023.104579

[47] Zhang C Du M Liao L Li W. The effect of air pollution on migrants' permanent settlement intention: evidence from China. J Clean Prod. (2022) 373:133832. 10.1016/j.jclepro.2022.13383235055496

[48] Nie P Peng X Luo T. Internet use and fertility behavior among reproductive-age women in China. China Econ Rev. (2023) 77:101903. 10.1016/j.chieco.2022.101903

[49] Liao L Kong S Du M. The effect of clean heating policy on individual health: evidence from China. China Econ Rev. (2025) 89:102309. 10.1016/j.chieco.2024.102309

[50] Bao T Liang B Riyanto YE. Unpacking the negative welfare effect of social media: Evidence from a large scale nationally representative time-use survey in China. China Econ Rev. (2021) 69:101650. 10.1016/j.chieco.2021.101650

[51] Linlin W Wanyu H Yuting L Huimin Q Zhi L Qinchen J. Research on the mechanism of short video information interaction behavior of college students with psychological disorders based on grounded theory. BMC Public Health. (2023) 23:2256. 10.1186/s12889-023-17211-4 37974096 PMC10652505

[52] Zhang R Su Y Lin Z Hu X. The impact of short video usage on the mental health of elderly people. BMC Psychol. (2024) 12:1–15. 10.1186/s40359-024-02125-6 39482710 PMC11529554

[53] Qu D Liu B Jia L Zhang X Chen D Zhang Q. The longitudinal relationships between short video addiction and depressive symptoms: a cross-lagged panel network analysis. Comput Human Behav. (2024) 152:108059. 10.1016/j.chb.2023.108059

[54] Abulibdeh ES Alneyadi S Skaik H El-Saleh MS Libdeh FIA Naser K. Short videoaddiction and its relationship with students' academic achievement and well-being: a pilot study. In: 2024 Global Digital Health Knowledge Exchange & *Empowerment Conference (gDigiHealth. KEE)*. Abu Dhabi: IEEE (2024). p. 1–6.

[55] Jerin SI O'Donnell N Mu D. Mental health messages on TikTok: analysing the use of emotional appeals in health-related# EduTok videos. Health Educ J. (2024) 83:395–408. 10.1177/00178969241235528

[56] Li J Liu Y. Intervention effect of the video health education model based on solution-focused theory on adolescents' mental health during the COVID-19 pandemic. Iran J Public Health. (2021) 50:2202. 10.18502/ijph.v50i11.7574 35223594 PMC8826342

[57] Ueda J Yamaguchi S Matsuda Y Okazaki K Morimoto T Matsukuma S. A randomized controlled trial evaluating the effectiveness of a short video-based educational program for improving mental health literacy among schoolteachers. Front Psychiatry. (2021) 12:596293. 10.3389/fpsyt.2021.596293 33716813 PMC7953138

[58] Winkler P. Janoušková M, Koženỳ J, Pasz J, Mladá K, Weissová A, et al. Short video interventions to reduce mental health stigma: a multi-centre randomised controlled trial in nursing high schools. Soc Psychiatry Psychiatr Epidemiol. (2017) 52:1549–57. 10.1007/s00127-017-1449-y 29101447

[59] Geschke D Lorenz J Holtz P. The triple-filter bubble: using agent-based modelling to test a meta-theoretical framework for the emergence of filter bubbles and echo chambers. Br J Soc Psychol. (2019) 58:129–49. 10.1111/bjso.12286 30311947 PMC6585863

[60] Ma X Sun Y Guo X Lai Kh Vogel D. Understanding users' negative responses to recommendation algorithms in short-video platforms: a perspective based on the Stressor-Strain-Outcome (SSO) framework. In: Electronic Markets. (2022). p. 1–18. 10.1007/s12525-021-00488-x

[61] Xiao Y Liu Z Wang B Zheng Y. The entertainment videos pushed by WeChat promote the mental health of undergraduate students. Heliyon. (2023) 9:e13776. 10.1016/j.heliyon.2023.e13776 36873492 PMC9981916

[62] Wu Y Wang X Hong S Hong M Pei M Su Y. The relationship between social short-form videos and youth's well-being: It depends on usage types and content categories. Psychol Popular Media. (2021) 10:467. 10.1037/ppm0000292

[63] Qin Y Omar B Musetti A. The addiction behavior of short-form video app TikTok: The information quality and system quality perspective. Front Psychol. (2022) 13:932805. 10.3389/fpsyg.2022.932805 36148123 PMC9486470

[64] Su C Zhou H Gong L Teng B Geng F Hu Y. Viewing personalized video clips recommended by TikTok activates default mode network and ventral tegmental area. Neuroimage. (2021) 237:118136. 10.1016/j.neuroimage.2021.118136 33951514

[65] Wen X Zhou Y Li Y Li X Qu P. Perceived Overload on Short Video Platforms and Its Influence on Mental Health Among the Elderly: A Moderated Mediation Model. Psychology Research and Behavior Management. (2024). p. 2347–2362. 10.2147/PRBM.S459426 38882234 PMC11179651

[66] Zhao Z Kou Y. Effects of loneliness on short video addiction among college students: the chain mediating role of social support and physical activity. Front Public Health. (2024) 12:1484117. 10.3389/fpubh.2024.1484117 39600403 PMC11588628

